# An Integrated Method for Tunnel Health Monitoring Data Analysis and Early Warning: Savitzky–Golay Smoothing and Wavelet Transform Denoising Processing

**DOI:** 10.3390/s23177460

**Published:** 2023-08-28

**Authors:** Ning Zhao, Jincheng Wei, Zhiyou Long, Chao Yang, Jiefu Bi, Zhaolong Wan, Shi Dong

**Affiliations:** 1 Key Laboratory of Highway Maintenance Technology Ministry of Transport, Jinan 250100, China; 2 Shandong Transportation Institute, Jinan 250100, China; 3 College of Transportation Engineering, Chang’an University, Xi’an 710064, China; 4 Engineering Research Center of Highway Infrastructure Digitalization, Ministry of Education of PRC, Chang’an University, Xi’an 710064, China; 5 Shaanxi Expressway Engineering Testing Inspection & Testing Co., Ltd., Xi’an 710086, China; 6 School of Highway, Chang’an University, Xi’an 710064, China

**Keywords:** Savitzky–Golay smoothing, wavelet transform denoising, tunnel health monitoring, system, early warning, coefficient of non-uniform variation

## Abstract

A tunnel health monitoring (THM) system ensures safe operations and effective maintenance. However, how to effectively process and denoise several data collected by THM remains to be addressed, as well as safety early warning problems. Thus, an integrated method for Savitzky–Golay smoothing (SGS) and Wavelet Transform Denoising (WTD) was used to smooth data and filter noise, and the coefficient of the non-uniform variation method was proposed for early warning. The THM data, including four types of sensors, were attempted using the proposed method. Firstly, missing values, outliers, and detrend in the data were processed, and then the data were smoothed by SGS. Furthermore, data denoising was carried out by selecting wavelet basis functions, decomposition scales, and reconstruction. Finally, the coefficient of non-uniform variation was employed to calculate the yellow and red thresholds. In data smoothing, it was found that the Signal Noise Ratio (SNR) and Root Mean Square Error (RMSE) of SGS smoothing were superior to those of the moving average smoothing and five-point cubic smoothing by approximately 10% and 30%, respectively. An interesting phenomenon was discovered: the maximum and minimum values of the denoising effects with different wavelet basis functions after selection differed significantly, with the SNR differing by 14%, the RMSE by 8%, and the r by up to 80%. It was found that the wavelet basis functions vary, while the decomposition scales are consistently set at three layers. SGS and WTD can effectively reduce the complexity of the data while preserving its key characteristics, which has a good denoising effect. The yellow and red warning thresholds are categorized into conventional and critical controls, respectively. This early warning method dramatically improves the efficiency of tunnel safety control.

## 1. Introduction

In recent years, highway tunnel diseases have become increasingly prominent, especially in tunnels with serious adverse geological locations, significant structural damage, mechanical fractures [1,2], or hidden dangers, which are prone to bridge breaks, tunnel collapse, mudslides, and other accidents [3,4,5]. These accidents can cause substantial economic and life losses. In addition, tunnels have high operational safety risks under complex geological conditions [6]. Establishing a health monitoring system for tunnels is an effective way to prevent disease and danger. It not only monitors settlement, cross-section, and surface stress in real-time but also reduces preventive maintenance costs [7,8]. Therefore, implementing a tunnel health monitoring (THM) system is imperative.

Some studies have been conducted to develop THM and analyze their data. Xie and Feng [9] discovered that the joint deformation of tunnel structure is the critical parameter for THM, based on an investigation of Shanghai power tunnels. Bossi et al. [10] described an Italian road THM and identified a deformation mechanism under landslide stress through monitoring data analysis. Yang et al. [11] adopted a multiple linear regression method to analyze THM data of an underwater Tunnel Boring Machine (TBM) tunnel. They proposed an early warning method for predicting the tunnel performance. Similar work was performed by Tan et al. [12]. Li et al. [13] developed a multi-layer deformation intelligent monitoring system for an inverted arch of salt rock tunnel, and applied it to the China–Laos Railway. However, TBM is inevitably affected by the environment, and sensors themselves, which can result in missing values, outliers, and noise in the data [14]. Such abnormal missing data and noise will affect monitoring in real-time and negatively impact the safety early warning [8]. Therefore, it is of great necessity to process and denoise the data of the THM.

Although tremendous research has been achieved in signal processing, and numerous studies have been conducted, some problems are found to exist in THM systems, such as missing values, outliers, trend terms, signal spikes, and signal noise in sampling signals. The current TBM signal processing and denoising methods include Wavelet Transform Denoising (WTD) processing, neural network, and other decomposition methods. Wang et al. [15] proposed a wavelet threshold denoising method to solve the data noise problem. This method can effectively reduce the interference of random noise. Fan et al. [16] developed a Residual Convolutional Neural Networks (ResNet) to denoise the vibration signal for the health monitoring system. They found ResNet not only removes noise from vibration signals but also preserves the most important vibration characteristics. Mousavi and Gandomi [17] used the Variational Mode Decomposition to denoise the signals and remove the seasonal patterns. The smoothing method is also a critical data processing step. Kaloop et al. [18] have performed smoothing and denoising by sliding average and wavelet transform. Their choice of wavelet basis functions and decomposition levels is not rigorous enough. Compared to these methods, the wavelet transform is the most effective denoising method [8]. In summary, they only conducted smoothing/denoising on the data without more thorough processing.

Currently, there is little research to deal with the above problems comprehensively and systematically. To achieve the goal of safety early warning for tunnel structures, all the mentioned problems significantly affect early warning research. Therefore, a more comprehensive, systematic signal processing method is proposed.

**Figure 1:**
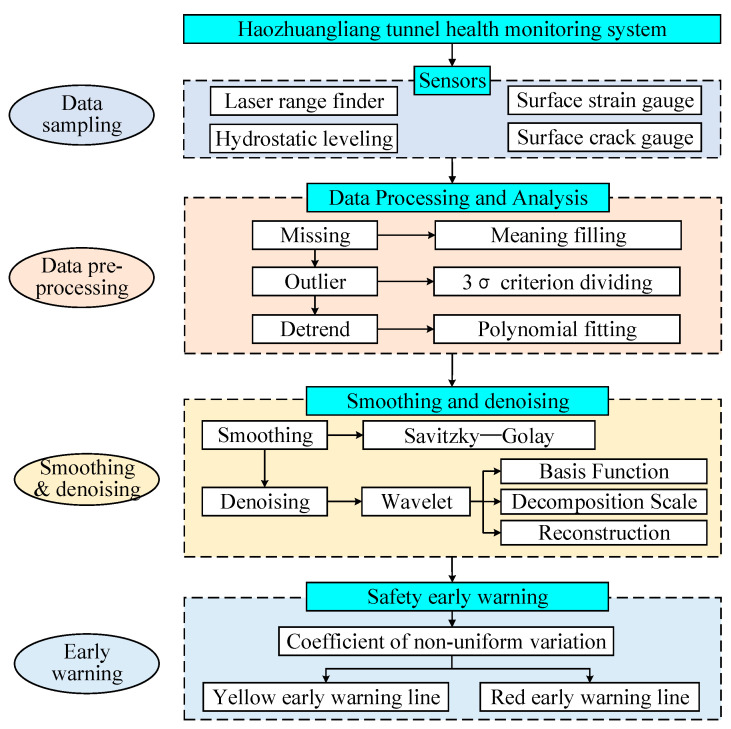
The flowchart of the organization for this study.

Firstly, for missing values, the average value of the time-series data is used for filling, assuming that small portions of data change do not affect the overall trend. Secondly, the 3σ rule is proposed to handle the outliers, indicating overly large or small data that need to be cleaned. Next, polynomial fitting is adopted to remove signal trend terms affected by seasonal and environmental factors. Then, in signal smoothing, Savitzky–Golay smoothing (SGS), moving average smoothing, and five-point cubic smoothing are used to smooth spikes existing in signals. In comparison, SGS is adopted when it shows better performance than others. Enumeration is utilized to select optimal parameters of SGS. Furthermore, to optimize wavelet denoising effects, wavelet transform is proposed to denoise data by selecting suitable wavelet basis functions and decomposition levels. Finally, the non-uniform variation coefficient method calculating the gray correlation degree between sensor signals is utilized. That is used to infer early warning situations of monitoring items. The flowchart of the organization for this study is shown in Figure 1.

The THM data for the Haozhuangliang tunnel in northwest China was used to validate the method. The sensors for health monitoring include hydrostatic levelling, laser range finder, surface strain gauge, and surface crack gauge. In the last step, the coefficient of the non-uniform variation method was adopted to provide safety early warning for tunnels.

## 2. Outlines of the Investigated Project

### 2.1. Outlines of the Haozhuangliang Tunnel

**Figure 2:**
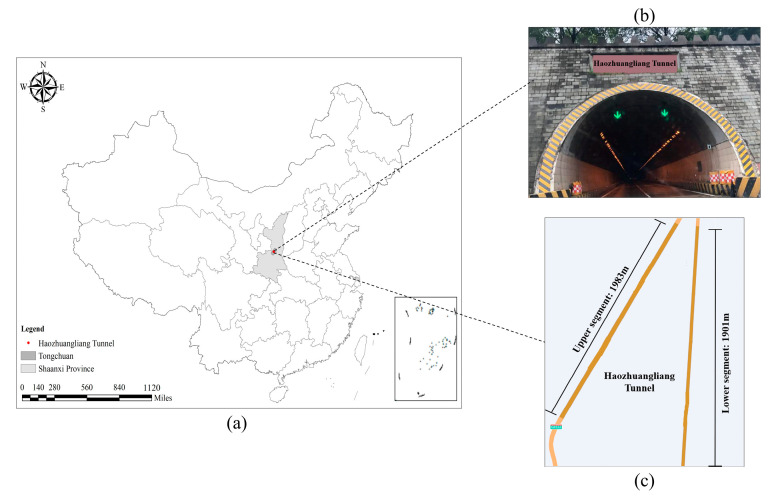
The location and composition of the Haozhuangliang tunnel. (**a**) Location and composition. (**b**) Haozhuangliang Tunnel. (**c**) Dimensions.

The Haozhuangliang tunnel is located on the Yan’an-Xi’an Expressway in Tongchuan, Shaanxi Province (Figure 2a), and is well equipped with ventilation, fire-fighting, and lighting facilities (Figure 2b). It is a dual-carriageway tunnel with four lanes in each direction. It is divided into two sections: (K125+230) to (K127+120) on the upper segment, and (K125+325) to (K127+060) on the lower part, respectively, measuring 1983 m and 1901 m (Figure 2c). Not only is it the longest completed high-grade highway tunnel in Shaanxi, but it also holds this distinction in Northwest China. The tunnel’s construction began officially in March 1998, and the entire line was opened to traffic on 29 April 2001.

### 2.2. Overview of THM System

The THM system for the Haozhuangliang tunnel deploys hydrostatic levelling (HL), laser range finder (LRF), surface strain gauge (SSG), and surface crack gauge (SCG) as the primary sensors, allowing the satisfaction of tunnel structure settlement and experimental test. The THM system provides (1) the dynamic monitoring and transmission of data, allowing the real-time status of the tunnel to be observed; (2) the highly accurate monitoring with a distance measurement accuracy of up to 0.5 mm; (3) the collection and storage of data, providing an early warning service when data falls outside of the acceptable range. The details of THM contents and sensors are listed in Table 1.

There are four types of monitoring: settlement, cross-section convergence, lining surface stress, and crack width. (1) Settlement monitoring. It is measured with hydrostatic leveling at nine monitoring sites every 200 m, with problematic sections located every 50 m. Data are collected once a minute, for 527,040 data points per year. (2) Cross-section convergence. A total of 32 laser range finders are deployed to monitor this content, with regular and problematic sections located at intervals of 200 and 50 m, respectively. The collection frequency is once an hour, with a total of 8041 data. (3) Lining surface stress. It is monitored using 131 surface strain gauges, with intervals following the same pattern as for settlement monitoring. There are five cross-sections per section, and 27 monitoring sites on the left and right sides. (4) Lining surface crack width. It is observed through 22 surface crack gauges, with one sensor selected for 25% of the total cracks. Data are collected once an hour for a total of 8785 data points.

**Figure 3:**
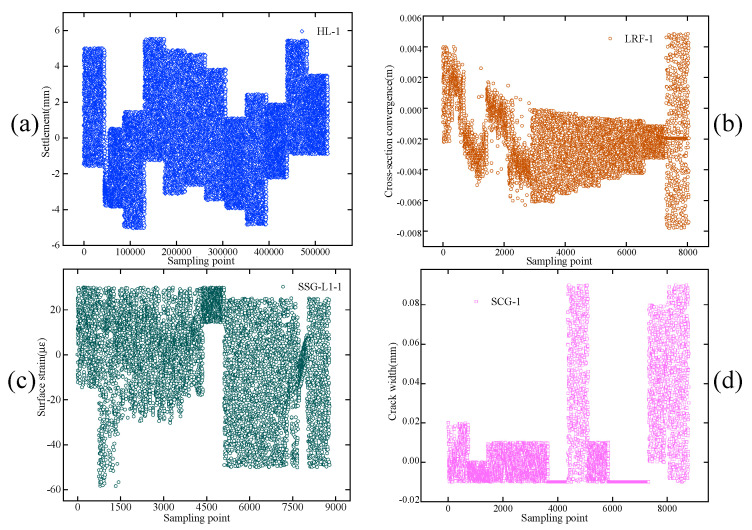
The one figure of the raw signals four types of THM sensors. (**a**) HL-1, (**b**) LRF-1, (**c**) SSG-L1-1 and (**d**) SCG-1.

We only show one figure of the raw signals collected from the THM for each sensor to repeat, as displayed in Figure 3. Figure 3a–d is a raw signals chart of HL-1, LDR-1, SSG-L1-1, and SCG-1, respectively.

## 3. Data Processing and Analysis

The four sensor types share similar data processing and analysis procedures in THM, and SSG is the most distributed sensor. Thus, this study employed SSG as a representative data processing and analysis example.

### 3.1. Data Processing

#### 3.1.1. Missing Value Processing

**Figure 4:**
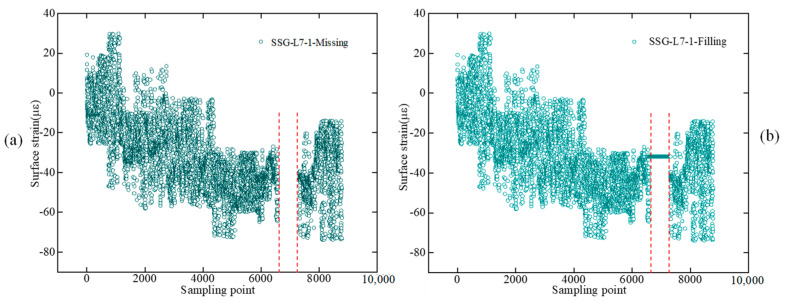
The missing data of SSG-L7-1 sensors. (**a**) the missing data, (**b**) the filling data.

Sensors may contain missing values due to various factors, such as power supply disruptions, maintenance checks, and other ambient factors. To facilitate further analysis, filling in these missing values is essential. The absence data of some sensors is appended in Table A1. Figure 4 shows the missing value of SSG-L7-1, “L” represents tunnel left, resulting in 744 missing values for each sensor. In comparison, SSG-R (SSG installed on the right side) exhibits no missing data, indicating that it has a higher quality of data concerning missing values. The leading causes of the missing data could be sensor failure or transmission network issues. This led to no data being collected for that month. The mean filling was utilized to fill in the missing values to reduce the amount of computational load and the effects of data variance. The filling result is shown in Figure 4, and the red dashed lines in Figure 4 indicate the extent of missing and filling, where (a,b) shows the missing and the filling of SSG-L7-1, separately.

#### 3.1.2. Outlier Value Processing

**Figure 5:**
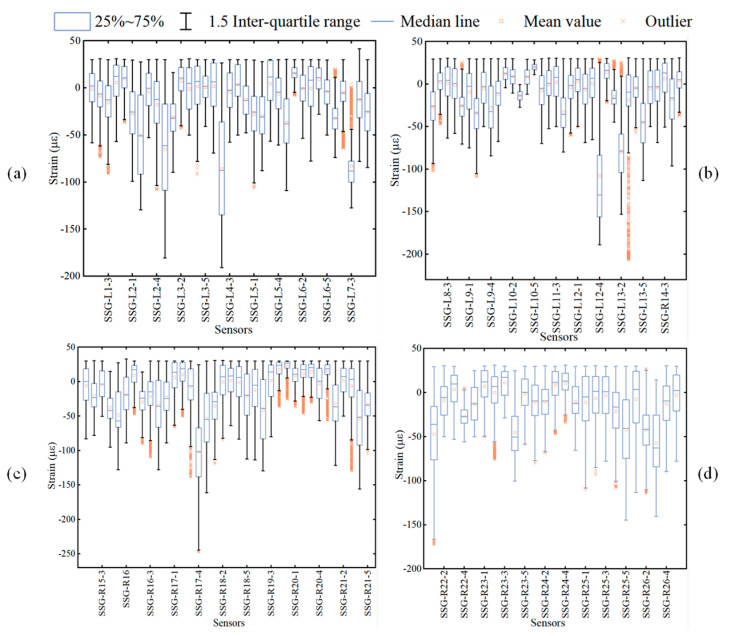
The box plot of SSG sensors. (**a**) S1–S7; (**b**) S7–S14; (**c**) S15–S21; and (**d**) S22–S26.

Data that deviate from overall sampling values are considered outliers due to problems in the signal transmission system or sensors, and conditions such as substantial electromagnetic interference. The presence of outliers can result in deviations in the data analysis. The outliers for some of the sensors are appended in Table A2. To address outliers, this study used standard methods such as the Pailda criterion (3σ rule) [19] and the Grubbs criterion [20]. The Grubbs criterion is complex and challenging to implement, and the 3σ criterion meets the requirements for outlier detection in THM. Therefore, the 3σ criterion is selected to remove the outliers in this study. The distribution of the tunnel SSG data is depicted by the box line diagram in Figure 5, in which it can be noted that the majority of SSG medians are centered around 0, with SSG-L2-2, SSG-L2-5, and SSG-L4-2 being exceptions having medians around −50. These sensors are mostly located at the top and waist of the vault, which are prone to stress changes. Multiple outlier values were observed for SSG-L13-3, SSG-R17-3, and SSG-R21-3, which have been replaced by the mean values.

#### 3.1.3. Detrending Processing

Seasonal changes in ambient factors or sensor performance can cause sensors to be susceptible to low-frequency noise. This phenomenon is known as the trend term of the signal, which requires removal for accurate signal analysis. A polynomial fitting is adopted to calculate the trend component of time-series data [21]. By subtracting the trend component from the original data, the detrended data can be obtained after removing the trend. In the process of detrending, the commonly used orders for polynomial fitting are 2–4 [22,23,24]. Based on the above research, a quadratic polynomial function was selected to predict the trend. Using an excessively high order can lead to overfitting, while a too low order results in insufficient fitting accuracy [25].

**Figure 6:**
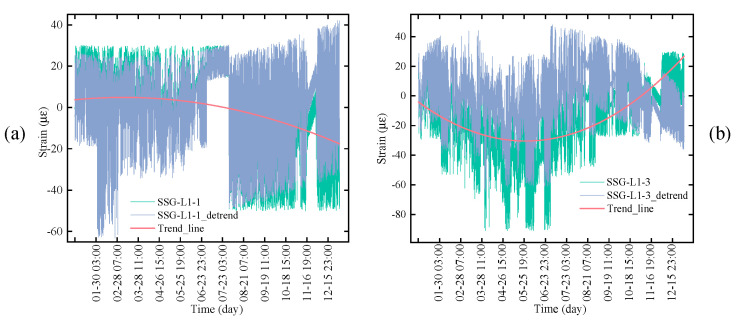
SSG-L1-1 and SSG-L1-1 detrending vs. original data. (**a**) SSG-L1-1, (**b**) SSG-L1-3.

The analysis showed that the two SSG data had a trend seasonality and residual, as shown in Figure 6. SSG-L1-1 (a) and SSG-L1-3 (b) data exhibit a decreasing trend from January to March and an increasing trend from June to September, possibly due to ambient factors, resulting in a noisy signal over time. Polynomial fitting was used to eliminate the trend item, as illustrated in Figure 6. SSG-L1-1 (a) and SSG-L1-3 (b) had different trend term changes. SSG-L1-1 was installed at the arch foot on the left lining, while SSG-L1-3 was at the arch crown. The various installation positions are the main reason for the differing trend changes.

### 3.2. Savitzky–Golay Smoothing

#### 3.2.1. Signal Evaluation Indexes

Signal Noise Ratio (SNR) and Root Mean Square Error (RMSE) are commonly used signal single evaluation indexes. *SNR* is the ratio between signal and noise power. The larger the *SNR*, the better the data smoothing. The equation of *SNR* [26] is defined as equation (1).

(1) $$SNR=10\log_{10}\left(\frac{\sum_{n}f^{2}(i)}{\sum_{n}{\left|f(i)-s(i)\right|}^{2}}\right)$$

*RMSE* is another index used to evaluate the denoising performance of the signal. It represents the square root of the variance of the smoothed data from the original data [27]. A lower *RMSE* indicates better denoising performance.

(2) $$RMSE=\sqrt{\frac{1}{n}\sum_{i=1}^{n}{\left[f(i)-s(i)\right]}^{2}}$$

where s(i) and f(i) denote the denoised and original signals, respectively, and n collection data point.

#### 3.2.2. Introduction of Savitzky–Golay Smoothing

Sensor data may contain signal glitches and not be smooth due to external factors such as manual operation and environmental effects. These interference signals will negatively impact monitoring and analysis. The data smoothing technique of numerical averaging is frequently utilized to mitigate the impact of external factors and simplify data analysis, enabling more precise visualization and summary of long-term trends.

Savitzky–Golay smoothing (SGS) was initially proposed by Savitzky and Golay in 1964 [28]. It is based on a local polynomial with least-squares fitting by moving windows, retaining most original data information while providing smoother distribution. The SGS [29,30] process is listed as follows:

(3) $${\hat{y}}_{j}=\frac{\sum_{i=-m}^{m}a_{i}x_{j+i}+a_{0}}{n}$$

where ${\hat{y}}_{j}$ denotes the smoothed data set, $x_{j+i}$ the collected data set, $a_{0}$ and $a_{i}$ the smoothing coefficients (e.g., the weight of $x_{j+i}$ in the smoothing window of period *i*), *n* the number of data in the sliding window. *m* the window width, and n=2m+1.

#### 3.2.3. Signal Smoothing

**Figure 7:**
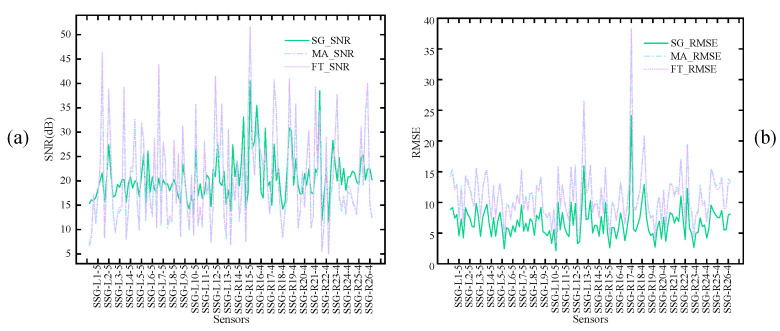
The SNR and RMSE line chat for SSG with three signal smoothing procedures. (**a**) the SNR of data smoothing, (**b**) the RMSE of data smoothing.

To demonstrate the superiority and applicability of SGS, we selected the moving average smoothing (MAS) [31] and five-point cubic smoothing (FTS) [32], which are relatively common in time-series data smoothing methods for comparison. And we used some indexes, including SNR and RMSE, to evaluate the smoothing effects. The SNR and RMSE for evaluating the smoothing impact are displayed in Figure 7a,b, respectively. And the two sensor smoothing results of SSG-L1 and SSG-R26 are shown in Table 2. It can be clearly compared that the SNR of MAS and FTS is distinctly smaller than that of SGS, and the RMSE of SGS is more significant than that of MAS and FTS. The main reason is that SGS allows direct specification of smoothing window sizes, making it suitable for signals of different frequencies. In contrast, the window sizes are fixed for FTS and MAS, which lacks flexibility. In addition, by calculating the mean values of SNR and RMSE across all sensors, it was found that the SNR and RMSE of SGS smoothing were superior to those of MAS and FTS by approximately 10% and 30%, respectively.

**Figure 8:**
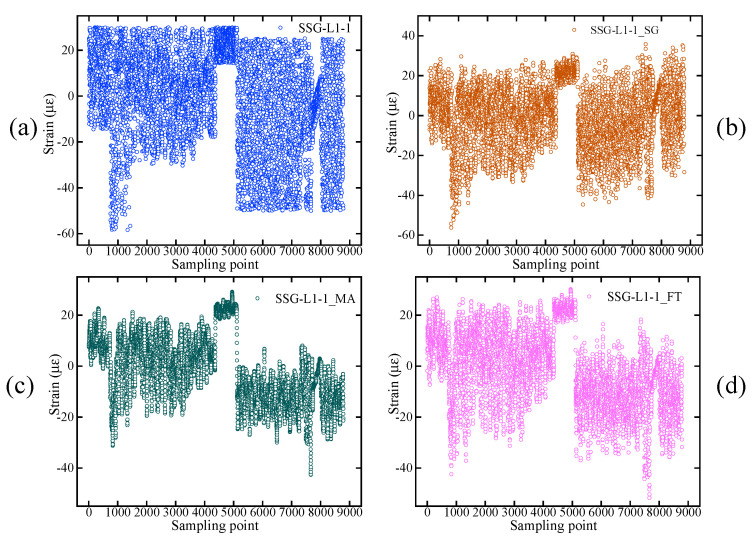
The result of four types of signal smoothing methods for SSG-L1-1. (**a**) the raw data, (**b**) SGS data, (**c**) MAS data, (**d**) FTS data.

Figure 8 shows three smoothing results, with Figure 8a–d representing the raw data, SGS, MAS, and FTS results, respectively. Compared to the raw data, SGS data does not lose too much detail, while MAS and FTS data differ considerably from the original data, such as in the signal sampling points (0, 1000) and (7000, 8000).

The fluctuation ranges of SSG vary across different periods, as shown by changes in smoothed data over the months. The data fluctuate between −20 and 20 με for most of the sampling points from 0 (around January) to 5000 (around August). However, there are more variations from −60 to 20 με between point 500 or so (February) and 1500 or so (around March). From point 5000 (August) to 8600 (December), the data range lies primarily between −40 με and 20 με. The effectiveness of SGS in preserving data features such as trends and fluctuations, further eliminating overlap and stacking, is clearly evident.

**Figure 9:**
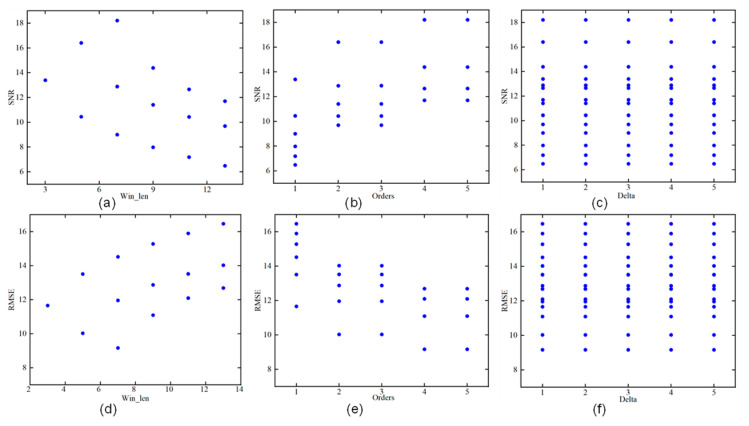
The relationship between mono-parameter and individual indicator. (**a**) SNR for different win_len, (**b**) SNR for different orders, (**c**) SNR for different delta; (**d**) RMSE for different win_len, (**e**) RMSE for different orders, (**f**) RMSE for different delta.

**Figure 10:**
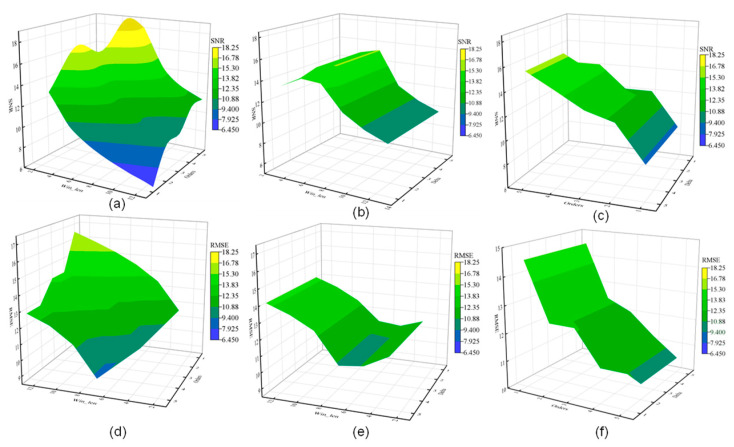
The relationship between multi-parameter and individual indicator. (**a**) SNR for different win_len and orders; (**b**) SNR for different win_len and delta; (**c**) SNR for different orders and delta; (**d**) RMSE for different win_len and orders; (**e**) RMSE for different win_len and delta; (**f**) RMSE for different orders and delta.

There are three standard parameters in the SGS procedures, including window length (Win_len), fitting order (Orders), and sample interval (Delta). After reading some relevant literature [30,31,33,34,35], we used an enumeration method to list every combination of parameters to smooth the data. We take some combination of parameters with Win_len (3–11, must be odd), Orders (1–5), and Delta (1–5). SNR and RMSE are used to evaluate the smoothing effect. The relationship between parameters and evaluation metrics is shown in Figure 9 and Figure 10. Figure 9a–c and Figure 9d–f shows the SNR and RMSE values of SGS for different window lengths, fitting orders, and sample intervals, respectively, and Figure 10a–c and Figure 10d–f shows the SNR values and RMSE values for window length and order, window length, and sample interval, and order and sample interval SG smoothing, respectively. The blue dots in Figure 9 represent the SGS processed values for different parameters. The smoothing results clearly indicate that too large a number results in poorly spaced samples in this study. The insensitivity of the sample interval to the SGS effect is primarily attributed to the fact that the sample interval commonly utilized does not differ considerably. Therefore, we obtained a simple Table 3 after removing the sample interval.

As is shown in Table 3, where the index includes window length and orders, the smoothing effects of parameter combination (Win_len = 7, Orders = 5) are better than others. The bolded portion of the table indicates the parameter with the best results, and this applies to the subsequent tables as well. The changes in window length are more sensitive than the fitting orders, and the reason is that the window length directly controls how many data points are included in each smoothed value. A longer window has more points and smooths more aggressively. An interesting phenomenon was discovered. When the window length of SG smoothing was more prominent than or equal to 5, the smoothing effects of fitting orders 2 and 3 did not differ much. This could be because the fitting order of the data was around 2–3. It was also similar to the polynomial fitting order in Section 3.1.3.

### 3.3. WTD Processing

#### 3.3.1. Introduction of WTD Processing

The coupling effect of various factors often affects the tunnel structure, including traffic behavior and ambient conditions. Data transmission inevitably results in signal quality degradation, leading to significant noise in time-domain data. Thus, noise reduction processing is necessary for the data. Fourier transform for time-domain analysis and wavelet transform for time-frequency analysis are typical denoising processes. The Fourier transform is unsuited for non-stationary, non-linear signals and is less sensitive to time changes [36]. On the other hand, wavelet transform (WT) can handle both non-smooth and noisy signals effectively [37]. Wavelet transform can combine the benefits of time-domain and frequency-domain analysis methods and characterize the local features of the signal in time-frequency analysis. It is frequently used to denoise tunnel monitoring data [8,38,39]. The results demonstrate the feasibility and effectiveness of applying wavelet transform to tunnel monitoring data processing. Based on these, this study will employ wavelet transform to reduce noise.

WT decomposes the raw signal into approximation and detail coefficients (*cD*). The decomposition process is stopped by discarding the low-frequency approximation coefficients (*cA*) from the early components and further decomposing the high-frequency detail coefficients until the denoising requirements are met. The signal is then reconstructed by retaining the high-frequency detail coefficients from each component and the approximation coefficients from the final components. This achieves the denoising effect on the signal. Therefore, the mathematical relationship between the wavelet reconstructed signal and its *cA* and *cD* [8,40] is shown in Equation (4) as follows:

(4) $$s(i)=cA_{n}+\sum_{j=1}^{n}cD_{j}$$

where s(i) is the denoised signal, $cA_{n}$ and $cD_{j}$ the approximation coefficients and the detail coefficients from the *n*-th and *j*-th decomposition, respectively.

#### 3.3.2. Evaluation Index of WTD Processing

In signal denoising processing, pursuing only the increase in SNR may result in over-denoising and the loss of valuable information [41]. To avoid this problem, the smoothness metric is introduced as a signal evaluation index. Smoothness (*r*) directly reflects the stability and continuity of the denoising signal, and can evaluate the preservation of the intrinsic characteristics of the signal after denoising [42]. It is a more intuitive evaluation metric. The combination of the smoothness metric with SNR and RMSE metrics allows for a more comprehensive and objective judgment of the effects of denoising algorithms.

Combining the smoothness metric with SNR and RMSE metrics allows for a more comprehensive and objective judgment of the effects of denoising algorithms [43]. *r* is the ratio of the root of variance between the denoised and original signal of first-order difference. It is a physical quantity concerned with signal approximation information [42,44]. The smaller the *r*, the better denoising.

(5) $$r=\frac{\sum_{n-1}{\left|s(i+1)-s(i)\right|}^{2}}{\sum_{n-1}{\left|f(i+1)-f(i)\right|}^{2}}$$

where s(i) and f(i) denote the denoised and original signals, respectively, and *n* collection data point.

To solve the problem that the three evaluation indicators may have different decisions and unclear references [45], we introduced the coefficient of variation method to synthesize the weight ratio. In this study, the coefficient of variation weighting method was introduced to determine the optimal number of layers for wavelet decomposition, which objectively reflects the complexity of calculating the index. When an index is difficult to estimate, it will have a more significant coefficient of variation and be assigned a higher weight.

*T_j_* is used to evaluate wavelet basis function and decomposition scale selection. The calculation of *T_j_* is firstly normalized for each index according to the correlation with the smoothing result [44].

(6) $$PSNR_{j}=\frac{SNR_{j}-SNR_{\max}}{SNR_{\max}-SNR_{\min}}$$

(7) $$PRMSE_{j}=\frac{RMSE_{j}-RMSE_{\min}}{RMSE_{\max}-RMSE_{\min}}$$

(8) $$Pr_{j}=\frac{r_{j}-r_{\min}}{r_{\max}-r_{\min}}$$

The weights are calculated and linearly combined to obtain the composite index *T_j_*: the smaller the *T_j_*, the better the wavelet decomposition [44,45].

(1) Calculate the coefficient of variation among the indexes $CV_{SNR}$.

(9) $$CV_{SNR}=\frac{\sigma_{SNR}}{\mu_{SNR}}$$

(2) Calculate the weights assigned to each index $W_{SNR}$.

(10) $$W_{SNR}=\frac{CV_{SNR}}{CV_{SNR}+CV_{RMSE}+CV_{r}}$$

(3) *T_j_* is obtained by linear combination.

(11) $$T_{j}=W_{SNR}\cdot PSNR_{j}+W_{RMSE}\cdot PRMSE_{j}+W_{r}\cdot Pr_{j}$$

where $\sigma_{SNR}$ and $\mu_{SNR}$ represent the variance and mean of the *SNR* series, respectively.

#### 3.3.3. Selection of Wavelet Basis Function

**Figure 11:**
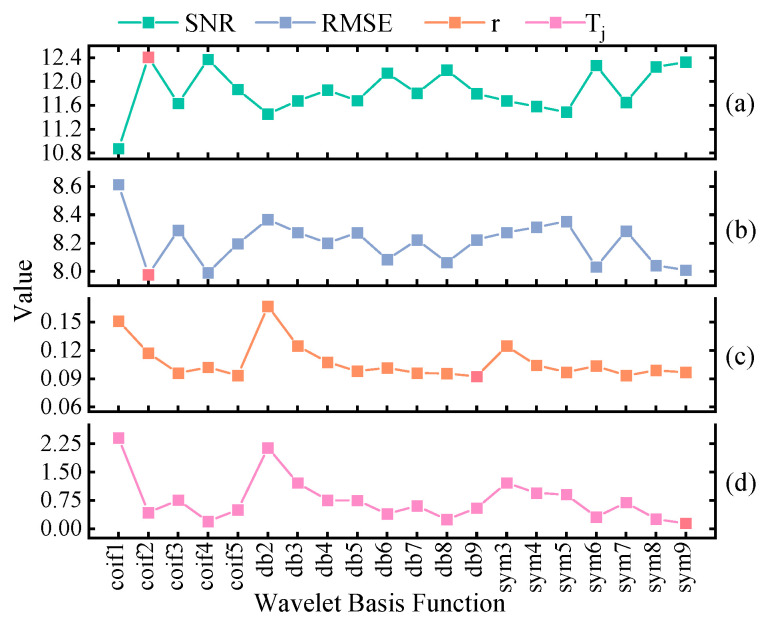
The evaluation of wavelets with different basis functions for SSG. (**a**) SNR, (**b**) RMSE, (**c**) r, (**d**) *T_j_*.

The selection of the wavelet basis function is a crucial factor in wavelet noise reduction, as each wavelet basis function has a unique effect on wavelet decomposition. Commonly used wavelet basis functions include coif1-5, db2-9, and sym3-9. This study compared the performance of coifN, dbN, and symN wavelet basis functions using an evaluation of Section 3.3.2, as presented in Figure 11a–d. Figure 11 depicts the variation values of the four metrics for evaluating the noise reduction effect of the wavelet transform under different wavelet basis functions, where the horizontal coordinates represent the different wavelet basis functions. Specifically, the green line represents the *SNR* in Figure 11a, the blue line represents the *RMSE* in Figure 11b, the orange line represents *r* in Figure 11c, and the pink line represents *T_j_* in Figure 11d. The red part of the line indicates the best basis function. Decomposition using coif2 produces the highest *SNR* and smaller *RMSE*. The *r* index of the db9 is smaller than other functions. It is particularly important to note that the decomposition using sym9 obtained the smallest *T_j_* index, with higher *SNR* and smaller *RMSE* and *r*. The red points in the figure indicate that the best noise reduction is achieved in the wavelet basis function for the transverse coordinate. Based on these results, sym9 was selected as the wavelet basis function for subsequent wavelet noise reduction. There is an interesting phenomenon that the maximum and minimum values of the denoising effects after selection differed greatly, with the SNR differing by 14%, the RMSE by 8%, and the r by up to 80%. This was mainly because different wavelet basis function clusters could fit the original function, but the detail parts were not very similar.

#### 3.3.4. Determination of Wavelet Decomposition Scale

The choice of wavelet decomposition scale (number of layers) is also an important factor affecting wavelet noise reduction. A high decomposition scale may filter out the local response signal, while a low decomposition scale may retain some of the noise, leading to suboptimal results. In quite a few studies [46,47,48], we learn that the decomposition scale is mainly chosen based on the data, but most of them choose below 7. As the number of decomposition layers continues to increase, the amplitude of the wavelet coefficients of the useful signal remains almost unchanged [40], implying that the noise reduction is no longer improving. In this paper, a range of 2 to 7 layers was selected as different sensor wavelet decomposition scales. The signal from each decomposition layer was assessed using a comprehensive evaluation index *T_j_*, and the results are shown in Table 4. HL, LRF, SSG, and SCG have the smallest index *T_j_* at a decomposition scale of 3 layers, which means that the optimal noise reduction effect occurs at 3 layers.

**Figure 12:**
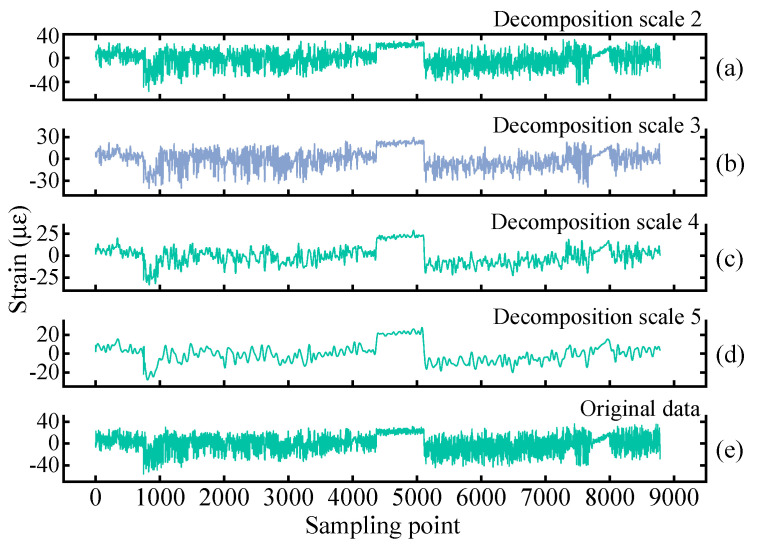
Different decomposition scales of SSG. (**a**) level 2, (**b**) level 3, (**c**) level 4, (**d**) level 5, (**e**) the original data.

To further support the decomposition scale of 3 layers from the data distribution perspective, plots of the decomposition results for the original SSG data at 2, 3, 4, and 5 decomposition layers are depicted in Figure 12a–e. The data at 3 layers does not suffer from the issue of retaining a significant amount of noise signals when decomposing 1 and 2 layers, nor does it over-filter local signals when decomposing from 4 and 5 layers. Therefore, a decomposition scale of 3 layers not only yields a better noise reduction performance for the original signal, but also preserves the overall variation trend.

#### 3.3.5. Reconstruction of the Wavelet Decomposition

**Figure 13:**
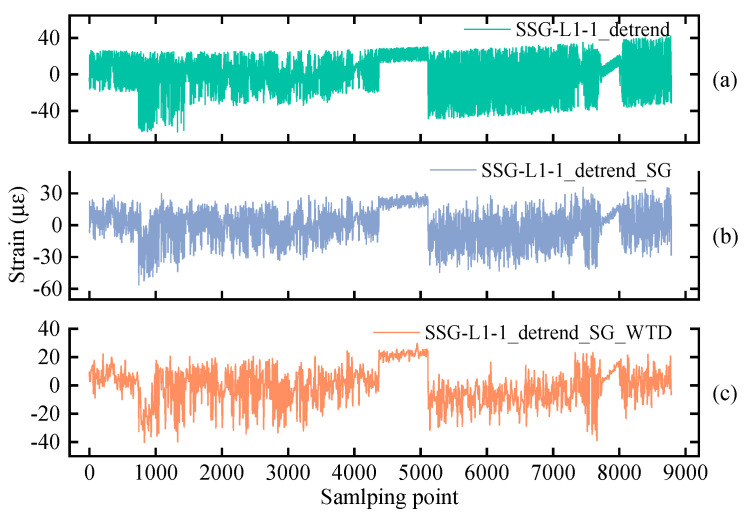
The result of SSG-L1-1 WTD. (**a**) the detrending data, (**b**) the SGS data, and (**c**) the WTD data.

The selected wavelet basis functions and decomposition scale decomposes the original signal into its corresponding wavelet coefficients. The threshold compromise function was selected in this study to threshold the wavelet coefficients, followed by their reconstruction to obtain the denoised wavelet signal. The results of detrending, SGS and denoising are illustrated in Figure 13. By comparing Figure 13a,b,c, it can be obtained that an integrated method of SGS and WTD can effectively remove noise better than SGS and detrending, while preserving the characteristics of the original data. Moreover, the reconstructed data are essentially consistent with the measured data’s trend and singular point location. This suggests that the integrated method has notable benefits in reducing signal noise.

### 3.4. Summary of Data Processing and Analysis

For the processing of the data collected by HL, LRF, and SCG sensors for THM, the same process as above for the SSG was performed, and the results are shown in Table 5. The HL and LRF have no missing values, but the SCG has missing values. The other three types of sensor data identify outlier values but lack trending terms. Regarding the selected optimal wavelet basis function, coif4, sym8, and db4 were respectively utilized for LRF, HL, and SCG data. This demonstrates that choosing the most suitable wavelet basis function for each sensor data is necessary when reconstructing wavelet decomposition.

## 4. Safety Early Warning

Determining the monitoring of early warning thresholds for each sensor is integral to the THM. Thus, this study introduced an early warning safety index for tunnels based on data processing and denoising, as presented in Section 4.1. The red and yellow early warning lines were calculated with sensors SSG as an example, and later extended to other sensors.

### 4.1. Selection of Early Warning Index

In early warning of changes in THM, the coefficient of non-uniform variation (CNV), which reflects the degree of curve similarity on the grey correlation analysis method, is used to display the degree of non-uniform variation of sensors at different locations [49,50]. The CNV is able to acquire the degree of correlation between the indexes. The CNV can give the degree of intercorrelation between different indicators. The larger the correlation degree, the greater the correlation between the current and standard data sequences. If the correlation degree is small, it indicates that non-uniform changes have occurred between the displacement sensors, non-uniform changes have occurred in the tunnel structure, accumulated damage may exist in THM, or there may be sensor failures [51]. Such non-uniform variations could display the presence of accrued damage or sensor faults. The CNV employs the slope correlation index, which is calculated as follows [49,50]:

(12) $$r(X_{0},X_{i})=n-1\sum_{k=1}^{n-1}\frac{1}{1+\left|\frac{a^{\left(1\right)}(x_{0}(k+1))}{x_{0}(k+1)}-\frac{a^{\left(1\right)}(x_{i}(k+1))}{x_{i}(k+1)}\right|}$$

(13) $$a^{\left(1\right)}(x(j+1))=x(j+1)-x(j)$$

where $x_{0}(i)$ and $x_{i}(i)$ denote the standard series and current compared series, respectively, $a^{\left(1\right)}(x_{i}(j))$ the cumulative reduction of the series x(j).

### 4.2. Evaluation of Safety Early Warning

**Figure 14:**
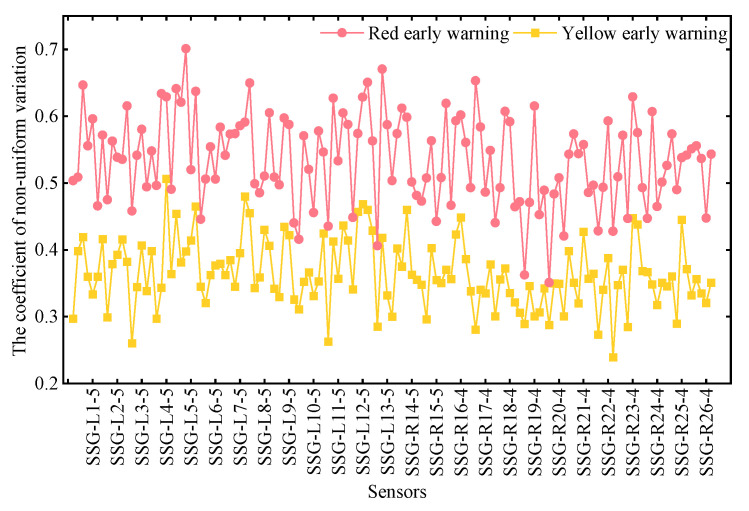
Early warning points for different SSG sensors.

The CNV in Section 4.1 was adopted to delineate the safety thresholds; yellow and red early warning thresholds were then defined. When the THM sensors reach the yellow early warning threshold, management and maintenance departments must pay attention to the tunnel environment, loads, and overall structural condition and arrange maintenance during this period. Suppose the monitoring values exceed the red early warning threshold. In that case, the management and maintenance departments need to pay immediate and significant attention by arranging an inspection and assessment of the tunnel’s structural safety to ensure its operation. Early warning thresholds differ from sensors of the same type located in different tunnel locations. Therefore, this study divided the early warning thresholds of all THM sensors in the tunnel and used SSG as an example. Figure 14 shows the early warning thresholds for each SSG sensor. The red early warning thresholds for the CNV mostly fall between 0.55 and 0.50, ranging from 0.35 to 0.7. Yellow early warning thresholds range between 0.3 and 0.4, ranging from 0.23 to 0.51. Consequently, the red early warning thresholds exhibit more significant variations across sensors than the yellow early warning thresholds.

**Figure 15:**
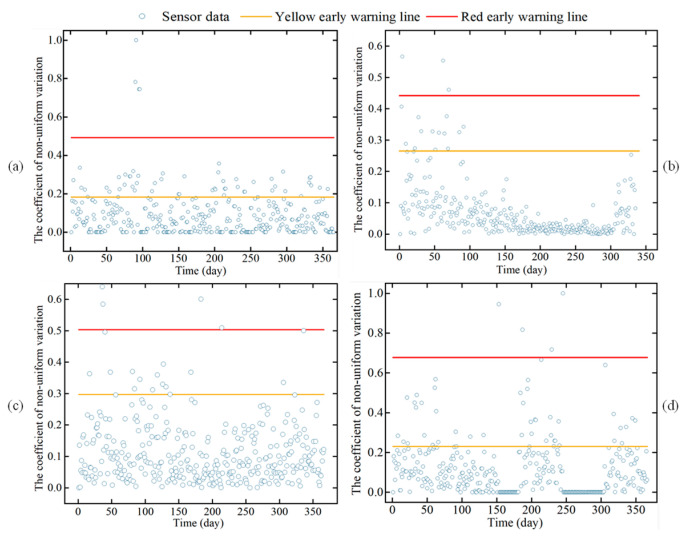
The early warning lines of four types of single sensors. (**a**) HL; (**b**) LRF; (**c**) SSG; (**d**) SCG.

One of the HL, LRF, SSG, and SCG was selected for early warning thresholds analysis on a day-by-day basis; the distribution for each sensor is shown in Figure 15a–d, respectively. It can be seen that most monitored values of the four sensors are within the safety threshold, with few reaching the yellow early warning line. This may be due to the vibration caused by heavy traffic in the tunnel on that day. On a few days, the monitored values reached the red early warning line, potentially due to extreme weather conditions, such as high winds and heavy rainfall. In such instances, it is crucial to dispatch personnel promptly for inspection and maintenance to avoid endangering the tunnel structure and ensure the safe operation of the tunnel. Furthermore, the Huangzhuangliang tunnel remains generally safe and experiences few safety hazards during daily inspections.

### 4.3. Validation

In order to verify the effectiveness of this method for tunnel health monitoring systems, we selected crack gauge data for comparative analysis. Since crack gauges measure the width of cracks in the tunnel, they best represent whether fatigue damage exists in the tunnel structure. Thus, we compared relevant Chinese standards: “*Technical Specifications for Maintenance of Highway Tunnels*” [52] and some literature studies [53,54]. Different warning levels of lining surface-crack width are divided, and the warning threshold for crack width is shown in Table 6.

**Figure 16:**
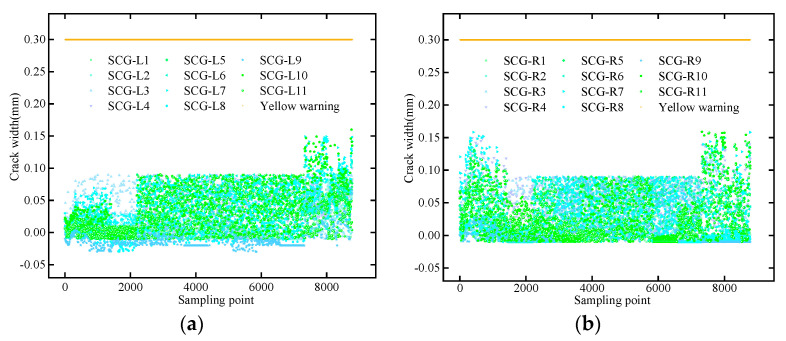
The early warning lines of SCG: (**a**) right tunnel monitoring for SCG; (**b**) left tunnel monitoring for SCG.

As shown in Figure 16a,b, we can clearly see the differences between the yellow warning line and the monitoring data before the early warning. This means that all the monitoring data from SCG of THM has not reached the warning value, indicating that the tunnel’s structural damage has not reached the warning line. Therefore, the early warning method we proposed has been proven to be practical and feasible.

## 5. Discussion

The signals sampled by the sensors in the THM system acquisition process are affected by various factors, such as environmental loads, material aging, and human traffic behaviors, resulting in the sampled signals containing multiple components. In addition, the operation of the sensors, the stability of the transmission networks, and the continuity of power and energy supply also greatly affect the quality of the acquired signals. In order to research structural safety warnings, the structural response signals need to be extracted from the complex signals. It is an important factor in evaluating the safe operation of tunnel structures. Thus, signal processing methods based on SGS and WTD have been proposed. However, during signal processing, the raw signals contain many interfering factors such as missing values, outliers, trend items, signal spikes, low-frequency noises, etc. To address these influencing factors, corresponding methods have been proposed, including filling missing values with the means, removing trend items using 3σ rule, smoothing out signal spikes using Savitzky–Golay smoothing, and reducing noises in the signals by wavelet transforms. There are some limitations and strengths of those methods as follows.

Firstly, in filling missing values, the mean filling is directly used to reduce computation and lower signal residuals, but we believe supervised machine learning methods could be used in future studies to predict missing values. Secondly, in outlier handling, using only the 3σ rule may introduce some bias, in other words, the defined quantiles for time-series data with large fluctuations may not be the same. Next, in detrending, the parameters of polynomial regression need to be further refined and scientized. Also, as an improved algorithm of least squares, SGS requires more delicate parameter tuning. Finally, in wavelet denoising, we propose combining adjustments of wavelet basis functions and decomposition scales to optimize the effect and using threshold shrinkage functions to constrain decomposition coefficients. This method shows apparent noise reduction effects and robustness. However, there is still room for optimization in parameter selection. Further studies may use particle swarm optimization (PSO) to search for optimum and achieve the best denoising outcomes globally. Currently, the assignment of parameters is still ambiguous, which makes it an intriguing and promising research direction.

For the study of tunnel safety early warnings, a method describing the interrelationships between sensors is proposed. By calculating the non-uniform variation coefficients of monitoring sensors across various categories in the THM system, changes in tunnel structural damages or monitoring errors between sensors can be observed, based on which the tunnel early warning situations are analyzed. Crack meters in the THM system are used to represent structural damage conditions to verify the feasibility of the non-uniform variation coefficient method, mainly because the width of lining cracks is the most direct and obvious damage precursor. However, the results show the crack widths do not reach the early warning threshold specified in the Chinese industrial standard “*Maintenance Technical Specifications for Highway Tunnels*”. Therefore, further validation of this method will be conducted on structures with significant damages or those reaching warning thresholds in future studies.

## 6. Conclusions

This study introduced the integration of SGS and wavelet transform for data processing and denoising. The coefficient of non-uniform variation (CNV) was then employed to determine the safety early warning threshold. According to the results, the following conclusions can be drawn:

(1) The problem on THM data for the Haozhuangliang tunnel, which is existing missing and outlier values, trend term, was addressed through filling or replacing with mean values, and polynomial fitting, respectively.
(2) Tunnels suffer from the impacts of multifaceted coupling effects such as traffic behavior and environment, and there is unavoidable signal loss during data transmission, which can lead to large amounts of noise being included in the data. Based on the effective preservation of data features, an integrated method of SGS and WTD can eliminate the issues of data overlap and stacked sections. By comparison, SGS is found to be better than the equivalent MAS and FTS. The mean values of SNR and RMSE of SGS smoothing were superior to those of MAS and FTS by approximately 10% and 30%, respectively.
(3) Three single THM evaluation indexes were coupled using the coefficient of variation method obtaining composite index *T_j_* to avoid too extreme evaluation results. For instance, selecting three layers for wavelet decomposition on four sensors is recommended when using index *T_j_*. However, recommendations for the other individual indexes may differ. Moreover, the maximum and minimum values of the denoising effects after selection differed greatly, with the SNR differing by 14%, the RMSE by 8%, and the r by up to 80%.
(4) A CNV method was proposed for safety early warning in the THM system, resulting in yellow and red early warning lines for the four sensors. The method enables daily monitoring of tunnel safety risks, and we validated them with data sampled by surface crack gauge.

This paper has provided a detailed description of the sampling data processing for THM. The health monitoring data from the Haozhuangliang Tunnel was used as a case study analysis. Processing was carried out respectively on missing values, outliers, trend terms, signal burrs, and signal noise. This fulfilled the goal of making up for the lack of comprehensive, systematic methods for dealing with tunnel health monitoring data. However, there are some regrets that the proposed method can be further improved, for example, more advanced methods can be adopted to predict missing values in missing value processing, the SGS parameter can also be optimized using neural networks or PSO algorithms, and more enriched data are still needed for early warning.

Compared with previous studies, this paper synthesizes several modules such as data preprocessing, SGS, WTD, and early warning studies in the THM system. Among them, the proposed SGS and WTD have good adaptability and robustness to the THM data, especially the parameter adjustment process of SGS and WTD. It is expected that more researchers will refer to the research method proposed in this paper to solve practical engineering problems.

## Figures and Tables

**Table 1:** Health monitoring contents and sensors.

Monitoring Contents	Sensors	Number	Model	Range	Unit
Settlement	HL	30	GSTP-YC11	±500	mm
Cross-section convergence	LRF	32	GSTP-DM-L1	30	m
Lining surface stress	SSG	135	GSTP-ZX300	±500	με
Lining surface crack width	SCG	25	GSTP-ZX540-12	±10	mm

**Table 2:** Three types of index of signal smoothing.

Sensors	SGS-SNR	SGS-RMSE	MAS-SNR	MAS-RMSE	FTS-SNR	FTS-RMSE
SSG-L1-1	15.335	8.832	6.670	14.598	7.140	14.259
SSG-L1-2	16.096	9.164	7.980	15.282	9.007	14.517
……	……	……	……	……	……	……
SSG-R26-4	22.483	7.984	14.504	13.797	15.494	13.130
SSG-R26-5	20.193	8.129	12.335	13.395	12.699	13.153

**Table 3:** SGS effects with different parameter combinations.

Indexes	(3, 1)	(5, 1)	(5, 2)	(5, 3)	(7, 1)	(7, 2)	(7, 3)	(7, 4)
SNR	13.393	10.445	16.405	16.406	8.997	12.879	12.884	18.206
RMSE	11.658	13.510	10.028	10.028	14.525	11.962	11.959	9.165
Indexes	**(7, 5)**	(9, 1)	(9, 2)	(9, 3)	(9, 4)	(9, 5)	(11, 1)	(11, 2)
SNR	**18.207**	7.981	11.408	11.411	14.386	14.389	7.187	10.430
RMSE	**9.164**	15.281	12.875	12.873	11.093	11.092	15.900	13.520
Indexes	(11, 3)	(11, 4)	(11, 5)	(13, 1)	(13, 2)	(13, 3)	(13, 4)	(13, 5)
SNR	10.435	12.653	12.657	6.488	9.693	9.698	11.699	11.701
RMSE	13.517	12.098	12.096	16.466	14.028	14.024	12.689	12.688

**Table 4:** *T_j_* for four sensors at different decomposition scales.

	Scale	1	2	3	4	5	6	7
Sensor								
HL		2.2355	0.8674	**0.8189**	0.8876	0.9340	0.9583	0.9707
LRF		1.8089	1.1921	**0.8770**	0.8914	0.9302	0.9577	0.9882
SSG		1.7259	1.2472	**0.9113**	0.9377	0.9687	0.9944	1.0117
SCG		1.8416	1.1975	**0.8746**	0.8903	0.9304	0.9563	0.9804

**Table 5:** Summary of data processing and denoising for four sensors.

Sensors	Data Processing			Wavelet Decomposition	
	Missing	Outlier	Trending	Basis Function	Decomposition Scale
HL	No	Yes	No	sym8	Three
LRF	No	Yes	No	coif4	
SSG	Yes	Yes	Yes	sym9	
SCG	Yes	Yes	No	db4	

**Table 6:** The warning threshold for crack width (mm).

Warning Level	Blue Level	Yellow Level	Origin Level	Red Level
Warning threshold	[0.3, 0.5]	[0.5, 2]	[2, 10]	[10, +∞]

## Data Availability

Data are available on request to the authors.

## Appendix

**Table A1:** The missing value of SSG sampled signals.

Time	SSG-L1-1	…	SSG-L7-1	SSG-L7-2	…	SSG-L10-1	SSG-L10-2	…	SSG-L13-1	SSG-L13-2	…
2020/1/1 0:00	0.117764	…	11.56338	−5.865	…	27.01734	22.23032	…	0.483888	−27.2743	…
2020/1/1 1:00	−8.88899	…	11.56338	−4.23563	…	27.5327	8.655122	…	0.960385	−28.1168	…
2020/1/1 2:00	25.1076	…	19.15	−7.5418	…	21.65632	5.698423	…	−0.26607	−27.5742	…
2020/1/1 3:00	22.53306	…	14.41916	−9.13067	…	19.57287	28.72267	…	−1.05225	−20.9412	…
2020/1/1 4:00	24.18905		4.152821	−13.8504		20.25839	27.52048		27.85579	16.96453	
…	…	…	…	…	…	…	…	…	…	…	…
2020/9/30 22:00	−23.4105		−45.3654	−2.25691		13.30216	1.166633		−26.406	−126.303	
2020/9/30 23:00	18.60308		−42.1273	−22.708		16.98568	1.02307		−19.5442	−128.158	
2020/10/1 0:00	−22.5817	…			…			…			…
2020/10/1 1:00	−33.7058	…			…			…			…
2020/10/1 2:00	−9.75376	…			…			…			…
…	…	…	…	…	…	…	…	…	…	…	…
2020/10/31 21:00	3.517012	…			…			…			…
2020/10/31 22:00	−2.70199	…			…			…			…
2020/10/31 23:00	−45.5726	…			…			…			…
2020/11/1 0:00	−3.17308	…	−41.5063	15.98343	…	−0.38406	−7.91329	…	−25.3404	−136.339	…
2020/11/1 1:00	−5.27697		−39.5268	14.80809		0.252836	−6.89927		−19.3655	−139.533	
…	…	…	…	…	…	…	…	…	…	…	…

**Table A2:** The outlier of SSG sampled signals.

Time	SSG-L1-1	…	SSG-L7-2	SSG-L7-3	…	SSG-L13-2	SSG-L13-3	…	SSG-L17-2	SSG-L17-3	…
2020/1/1 0:00	0.117764	…	−5.865	−4.74245	…	−27.2743	**−231.669 ***	…	4.442265	−28.7319	…
2020/1/1 1:00	−8.88899	…	−4.23563	−9.54641	…	−28.1168	**−185.268**	…	21.57386	−30.6786	…
2020/1/1 2:00	25.1076	…	−7.5418	−14.4818	…	−27.5742	**−173.823**	…	23.89546	−37.6942	…
2020/1/1 3:00	22.53306	…	−9.13067	−15.1492	…	−20.9412	**−191.015**	…	22.34036	−23.1502	…
2020/1/1 4:00	24.18905		−13.8504	−18.8737		16.96453	**−171.888**		26.19974	−32.0556	
…	…	…	…	…	…	…	…	…	…	…	…
2020/12/30 6:00	−30.73445		−13.27500	**−122.95452**		**−111.22073**	−24.063804		−16.142594	−53.7791791	
2020/12/30 7:00	−2.939612		−51.07349	**−108.05770**		**−142.77616**	−30.377180		−12.234906	−59.4873925	
2020/12/30 8:00	−25.88311	…	−42.29088	**−125.34516**	…	**−120.00010**	−26.886667	…	17.397010	−63.6768374	…
2020/12/30 9:00	−10.04477	…	−28.29517	**−108.70015**	…	**−138.90669**	−27.853262	…	−27.079540	**−78.5692583**	…
2020/12/30 10:00	−0.252702	…	13.267755	−69.065564	…	**−130.53009**	−22.418497	…	10.885415	**−85.71**	…
…	…	…	…	…	…	…	…	…	…	…	…
2020/12/31 15:00	18.374821	…	−53.53581	**−125.57384**	…	**−128.74272**	−23.872604	…	23.1728820	−40.1747286	…
2020/12/31 16:00	−47.68239	…	−44.71414	−77.952204	…	−89.493094	−22.914510	…	−24.769117	−40.0632853	…
2020/12/31 17:00	2.8328274	…	23.539159	−103.98833	…	−104.45871	−32.379206	…	−42.033104	−44.7867503	…
2020/12/31 18:00	−25.94758	…	−10.70891	−87.453930	…	−91.676369	−53.719138	…	−36.237575	−60.6308586	…
2020/12/31 19:00	7.2367973		−18.89661	**−126.77552**		−137.67549	−44.276915		2.78697635	−60.2144653	
…	…	…	…	…	…	…	…	…	…	…	…

* The bolded numbers stand for the outlier of the corresponding sensors.

## References

[1] Nasiri S., Khosravani M.R., Weinberg K. (2017). Fracture Mechanics and Mechanical Fault Detection by Artificial Intelligence Methods: A Review. Eng. Fail. Anal..

[2] Guan Q., Xu Y., Wang J., Wu Q., Zhang P. (2023). Meso-Scale Fracture Modelling and Fracture Properties of Rubber Concrete Considering Initial Defects. Theor. Appl. Fract. Mech..

[3] Zhong Z., Wang Z., Zhao M., Du X. (2020). Structural Damage Assessment of Mountain Tunnels in Fault Fracture Zone Subjected to Multiple Strike-Slip Fault Movement. Tunn. Undergr. Space Technol..

[4] Niu F., Cai Y., Liao H., Li J., Tang K., Wang Q., Wang Z., Liu D., Liu T., Liu C. (2022). Unfavorable Geology and Mitigation Measures for Water Inrush Hazard during Subsea Tunnel Construction: A Global Review. Water.

[5] Zhu Y., Zhou J., Zhang B., Wang H., Huang M. (2022). Statistical Analysis of Major Tunnel Construction Accidents in China from 2010 to 2020. Tunn. Undergr. Space Technol..

[6] Chen Z., He C., Yang W., Guo W., Li Z., Xu G. (2020). Impacts of Geological Conditions on Instability Causes and Mechanical Behavior of Large-Scale Tunnels: A Case Study from the Sichuan–Tibet Highway, China. Bull. Eng. Geol. Environ..

[7] Wang B., Zhang Z., He C., Zheng H. (2017). Implementation of a Long-Term Monitoring Approach for the Operational Safety of Highway Tunnel Structures in a Severely Seismic Area of China. Struct Control Health Monit.

[8] Jiang X., Lang Q., Jing Q., Wang H., Chen J., Ai Q. (2022). An Improved Wavelet Threshold Denoising Method for Health Monitoring Data: A Case Study of the Hong Kong-Zhuhai-Macao Bridge Immersed Tunnel. Appl. Sci..

[9] Xie X.Y., Feng L. (2012). Real-Time Health Monitoring System for Power Tunnel. Proceedings of the GeoCongress 2012.

[10] Bossi G., Schenato L., Marcato G. (2017). Structural Health Monitoring of a Road Tunnel Intersecting a Large and Active Landslide. Appl. Sci..

[11] Yang J.-P., Chen W.-Z., Li M., Tan X.-J., Yu J. (2018). Structural Health Monitoring and Analysis of an Underwater TBM Tunnel. Tunn. Undergr. Space Technol..

[12] Tan X., Chen W., Wu G., Wang L., Yang J. (2020). A Structural Health Monitoring System for Data Analysis of Segment Joint Opening in an Underwater Shield Tunnel. Struct. Health Monit..

[13] Li J., Zhou F., Zhou P., Lin J., Jiang Y., Wang Z. (2021). A Health Monitoring System for Inverted Arch of Salt Rock Tunnel Based on Laser Level Deformation Monitor and WFBG. Measurement.

[14] Yan Y., Mao X., Wang X., Yu X., Fang L. (2019). Design and Implementation of a Structural Health Monitoring System for a Large Sea-Crossing Project with Bridges and Tunnel. Shock Vib..

[15] Wang X., Huang S., Kang C., Li G., Li C. (2020). Integration of Wavelet Denoising and HHT Applied to the Analysis of Bridge Dynamic Characteristics. Appl. Sci..

[16] Fan G., Li J., Hao H. (2020). Vibration Signal Denoising for Structural Health Monitoring by Residual Convolutional Neural Networks. Measurement.

[17] Mousavi M., Gandomi A.H. (2021). Structural Health Monitoring under Environmental and Operational Variations Using MCD Prediction Error. J. Sound Vib..

[18] Kaloop M.R., Hussan M., Kim D. (2019). Time-Series Analysis of GPS Measurements for Long-Span Bridge Movements Using Wavelet and Model Prediction Techniques. Adv. Space Res..

[19] Xu J. Missing Data Imputation in Bridge Monitoring System Based on the Prediction Algorithms. Proceedings of the 2023 IEEE 2nd International Conference on Electrical Engineering, Big Data and Algorithms (EEBDA).

[20] Zhang X., Li J. (2019). Treatment of Errors in Dam Safety Monitoring Data. IOP Conf. Ser. Earth Environ. Sci..

[21] Kaya Y., Safak E. (2015). Real-Time Analysis and Interpretation of Continuous Data from Structural Health Monitoring (SHM) Systems. Bull Earthq. Eng.

[22] Yang B., Yin K., Lacasse S., Liu Z. (2019). Time Series Analysis and Long Short-Term Memory Neural Network to Predict Landslide Displacement. Landslides.

[23] Lei Y., Lin J., He Z., Zuo M.J. (2013). A Review on Empirical Mode Decomposition in Fault Diagnosis of Rotating Machinery. Mech. Syst. Signal Process..

[24] Wang Y., Xiang J., Markert R., Liang M. (2016). Spectral Kurtosis for Fault Detection, Diagnosis and Prognostics of Rotating Machines: A Review with Applications. Mech. Syst. Signal Process..

[25] Worden K., Cross E.J. (2018). On Switching Response Surface Models, with Applications to the Structural Health Monitoring of Bridges. Mech. Syst. Signal Process..

[26] Yang H., Cheng Y., Li G. (2021). A Denoising Method for Ship Radiated Noise Based on Spearman Variational Mode Decomposition, Spatial-Dependence Recurrence Sample Entropy, Improved Wavelet Threshold Denoising, and Savitzky-Golay Filter. Alex. Eng. J..

[27] Alyasseri Z.A.A., Khader A.T., Al-Betar M.A., Abasi A.K., Makhadmeh S.N. (2020). EEG Signals Denoising Using Optimal Wavelet Transform Hybridized with Efficient Metaheuristic Methods. IEEE Access.

[28] Savitzky A., Golay M.J.E. (1964). Smoothing and Differentiation of Data by Simplified Least Squares Procedures. Anal. Chem..

[29] Acharya D., Rani A., Agarwal S., Singh V. (2016). Application of Adaptive Savitzky–Golay Filter for EEG Signal Processing. Perspect. Sci..

[30] Sadeghi M., Behnia F., Amiri R. (2020). Window Selection of the Savitzky–Golay Filters for Signal Recovery from Noisy Measurements. IEEE Trans. Instrum. Meas..

[31] Jardim R., Morgado-Dias F. (2020). Savitzky–Golay Filtering as Image Noise Reduction with Sharp Color Reset. Microprocess. Microsyst..

[32] Yang C., Tong X., Chen G., Yuan C., Lian J. (2023). Assessment of Seismic Landslide Susceptibility of Bedrock and Overburden Layer Slope Based on Shaking Table Tests. Eng. Geol..

[33] Agarwal S., Rani A., Singh V., Mittal A.P. (2015). Performance Evaluation and Implementation of FPGA Based SGSF in Smart Diagnostic Applications. J. Med. Syst..

[34] Zhao A.-X., Tang X.-J., Zhang Z.-H., Liu J.-H. The Parameters Optimization Selection of Savitzky-Golay Filter and Its Application in Smoothing Pretreatment for FTIR Spectra. Proceedings of the 2014 9th IEEE Conference on Industrial Electronics and Applications.

[35] Ramakrishnan V., Pete D.J. (2021). Savitzky–Golay Filtering-Based Fusion of Multiple Exposure Images for High Dynamic Range Imaging. SN Comput Sci..

[36] Fang W., Shao Y., Love P.E.D., Hartmann T., Liu W. (2023). Detecting Anomalies and De-Noising Monitoring Data from Sensors: A Smart Data Approach. Adv. Eng. Inform..

[37] Lependin A.A., Ilyashenko I.D., Nasretdinov R.S. (2020). Use of Trainable Wavelet Transform with Adaptive Threshold Filtering for Noise Reduction of Speech Signals. J. Phys. Conf. Ser..

[38] Zhu J., Xue Y., Zhang N., Li Z., Tao Y., Qiu D. (2017). A Noise Reduction Method for Ground Penetrating Radar Signal Based on Wavelet Transform and Application in Tunnel Lining. IOP Conf. Ser. Earth Environ. Sci..

[39] Chen J., Jiang X., Yan Y., Lang Q., Wang H., Ai Q. (2022). Dynamic Warning Method for Structural Health Monitoring Data Based on ARIMA: Case Study of Hong Kong–Zhuhai–Macao Bridge Immersed Tunnel. Sensors.

[40] Li H., Shi J., Li L., Tuo X., Qu K., Rong W. (2022). Novel Wavelet Threshold Denoising Method to Highlight the First Break of Noisy Microseismic Recordings. IEEE Trans. Geosci. Remote Sens..

[41] Jiang X., Mahadevan S., Adeli H. (2007). Bayesian Wavelet Packet Denoising for Structural System Identification. Struct. Control Health Monit..

[42] Shao S., Wang T., Wang L., Li S., Yao C. A Photoplethysmograph Signal Preprocess Method Based on Wavelet Transform. Proceedings of the 2021 36th Youth Academic Annual Conference of Chinese Association of Automation (YAC).

[43] Halidou A., Mohamadou Y., Ari A.A.A., Zacko E.J.G. (2023). Review of Wavelet Denoising Algorithms. Multimed. Tools Appl..

[44] Shi G., Hu P., Chen J., Li C., Zhou H., Ma Y. Wavelet De-Noising Method Analysis of Pipeline Magnetic Flux Leakage In-Line Inspection Based on Coefficient of Variation. Proceedings of the 2022 International Conference on Automation, Robotics and Computer Engineering (ICARCE).

[45] Li Z., Shi W., Zhang H., Hao M. (2017). Change Detection Based on Gabor Wavelet Features for Very High Resolution Remote Sensing Images. IEEE Geosci. Remote Sens. Lett..

[46] Srivastava M., Anderson C.L., Freed J.H. (2016). A New Wavelet Denoising Method for Selecting Decomposition Levels and Noise Thresholds. IEEE Access.

[47] Pradhan P.S., King R.L., Younan N.H., Holcomb D.W. (2006). Estimation of the Number of Decomposition Levels for a Wavelet-Based Multiresolution Multisensor Image Fusion. IEEE Trans. Geosci. Remote Sens..

[48] Liu Y., Song Y., Zhang Y., Liao Z. (2022). WT-2DCNN: A Convolutional Neural Network Traffic Flow Prediction Model Based on Wavelet Reconstruction. Phys. A Stat. Mech. Appl..

[49] Gu H., Yang M., Gu C., Fang Z., Huang X. (2022). A Comprehensive Evaluation Method for Concrete Dam Health State Combined with Gray-Analytic Hierarchy-Optimization Theory. Struct. Health Monit..

[50] Huang J., Sun L., Meng H., Shi Z., Goertzel B., Feng J. (2017). Beam Bridge Health Monitoring Algorithm Based on Gray Correlation Analysis. Proceedings of the Intelligence Science I.

[51] Sun Y. (2018). Study on Early Warning and Safety Assessment of Bridge Structure Based on Safety Monitoring Data. Master’s Thesis.

[52] Chongqing Transportation Commission (2005). Technical Specification for Highway Tunnel Maintenance: JTG H12-2003.

[53] Liu Y., Yang E., Liu S. Detection of Railway Tunnel Lining Based on Adaptive Background Learning. Proceedings of the 2020 15th IEEE International Conference on Signal Processing (ICSP).

[54] Ang H., Ma F., Su J., Guo H., Li K. (2020). Development and Application of Tunnel Apparent Distress Monitoring System Based on Video Image Analysis. IOP Conf. Ser. Mater. Sci. Eng..

